# Higenamine Reduces Fine-Dust-Induced Matrix Metalloproteinase (MMP)-1 in Human Keratinocytes

**DOI:** 10.3390/plants12132479

**Published:** 2023-06-28

**Authors:** DongHyeon Kim, JeaHyeok Yun, Eunmiri Roh, Han-Seung Shin, Jong-Eun Kim

**Affiliations:** 1 Department of Food Science and Biotechnology, Dongguk University-Seoul, Goyang-si 10326, Republic of Korea; calbea@naver.com; 2 Department of Food Science and Technology, Korea National University of Transportation, Jeungpyeong 27909, Republic of Korea; qweasd126@naver.com; 3 Department of Cosmetic Science, Kwangju Women’s University, Gwangju 62396, Republic of Korea; roheunmiri@kwu.ac.kr

**Keywords:** higenamine, find dust, skin, air pollution

## Abstract

Environmental pollutants such as fine dust are increasingly linked to premature skin aging. In this study, we investigated the protective effects of higenamine, a natural plant alkaloid, against fine-dust-induced skin aging in human keratinocytes (HaCaT cells). We found that higenamine significantly attenuated fine-dust-induced expression of matrix metalloproteinase-1 (MMP-1), a key enzyme involved in collagen degradation. Furthermore, higenamine was found to modulate fine-dust-induced AP-1 and NF-κB transactivation, which are crucial factors for MMP-1 transcription. Higenamine also impeded fine-dust-induced phosphorylation in specific pathways related to AP-1 and NF-κB activation, and effectively alleviated reactive oxygen species (ROS) production, a key factor in oxidative stress caused by fine dust exposure. These results suggest that higenamine exerts protective effects against fine-dust-induced skin aging, primarily through its MMP-1 inhibitory properties and ability to mitigate ROS-induced oxidative damage. Our data highlight the potential of higenamine as an effective ingredient in skincare products designed to combat environmental skin damage.

## 1. Introduction

Particulate matter, also known as fine dust, consists of tiny particles suspended in the atmosphere, and it can pose significant health and environmental risks [1]. These particles primarily originate from anthropogenic activities such as vehicle exhaust, industrial plants, power generation facilities, and road construction, as well as natural phenomena such as volcanic eruptions and dust storms [2]. The primary constituents of fine dust include sulfates, nitrates, carbon compounds, and metals. The environmental implications of fine dust are noteworthy. As an atmospheric pollutant, it can degrade visibility due to light scattering and absorption. Furthermore, when it descends to the ground with precipitation, it contributes to water pollution and soil heavy metal contamination, consequently impacting ecosystems [2]. Fine dust can also exacerbate climate change issues, including global warming and acid rain [1].

Fine dust can cause severe health problems in humans. The smaller the particulate matter, the more readily it can infiltrate human lungs, elevating the risk of respiratory and circulatory diseases [1]. Symptoms can range from coughing, throat irritation, itchy eyes, and breathing difficulties to asthma attacks. Long-term exposure can lead to grave conditions such as cancer, heart disease, and stroke. In particular, fine dust negatively affects skin health. High concentrations of fine dust particles on the skin surface can cause damage that results in various skin problems [3].

Particulate matter, such as fine dust, adhering to the skin can induce inflammatory responses, resulting in erythema and potentially provoking sensations of itching or stinging [4,5]. Prolonged exposure may compound these skin irritations. Minute particles of fine dust can penetrate deeply into skin pores, obstructing them and impeding the expulsion of sebum and cellular waste products, which can cause the development of acne, pimples, and blackheads [6]. Moreover, the adherence of fine dust to the skin surface can compromise the integrity of the natural protective lipid barrier, leading to increased skin dehydration and heightened sensitivity [7]. Further exacerbating its deleterious effects, fine dust significantly contributes to skin aging [8]. It induces oxidative stress within the skin, which, in turn, promotes collagen degradation, thereby diminishing skin elasticity and facilitating the formation of wrinkles [7]. This cascade of events accelerates the skin aging process, potentially culminating in premature skin aging [9]. Therefore, fine dust not only presents immediate dermatological challenges but also contributes to long-term degradation of skin health.

Matrix metalloproteinase-1 (MMP-1) is an enzyme that plays a crucial role in the breakdown of the extracellular matrix (ECM) during physiological and pathological processes, including skin aging [10]. The ECM is a network of proteins and carbohydrates that provides structure and support to cells and tissues in the body [11]. In the skin, the ECM is primarily composed of collagen, elastin, and hyaluronic acid [10]. In youthful, healthy skin, there is a balance between the production of new ECM components (particularly collagen) and the breakdown of old or damaged components [12]. This balance ensures that the skin maintains strength, elasticity, and hydration. However, this balance can be disrupted with age and exposure to environmental factors such as UV radiation or pollution [13]. MMP-1, also known as collagenase, specifically targets and breaks down collagen, one of the main structural proteins in the skin. When MMP-1 activity is upregulated, as is often the case with UV exposure, more collagen is broken down than produced [14]. Over time, this leads to a net loss of collagen, resulting in visible signs of skin aging, such as wrinkles, reduced elasticity, and sagging [15]. Therefore, interventions that regulate MMP-1 activity may have potential applications in preventing or slowing skin aging.

**Figure 1:**
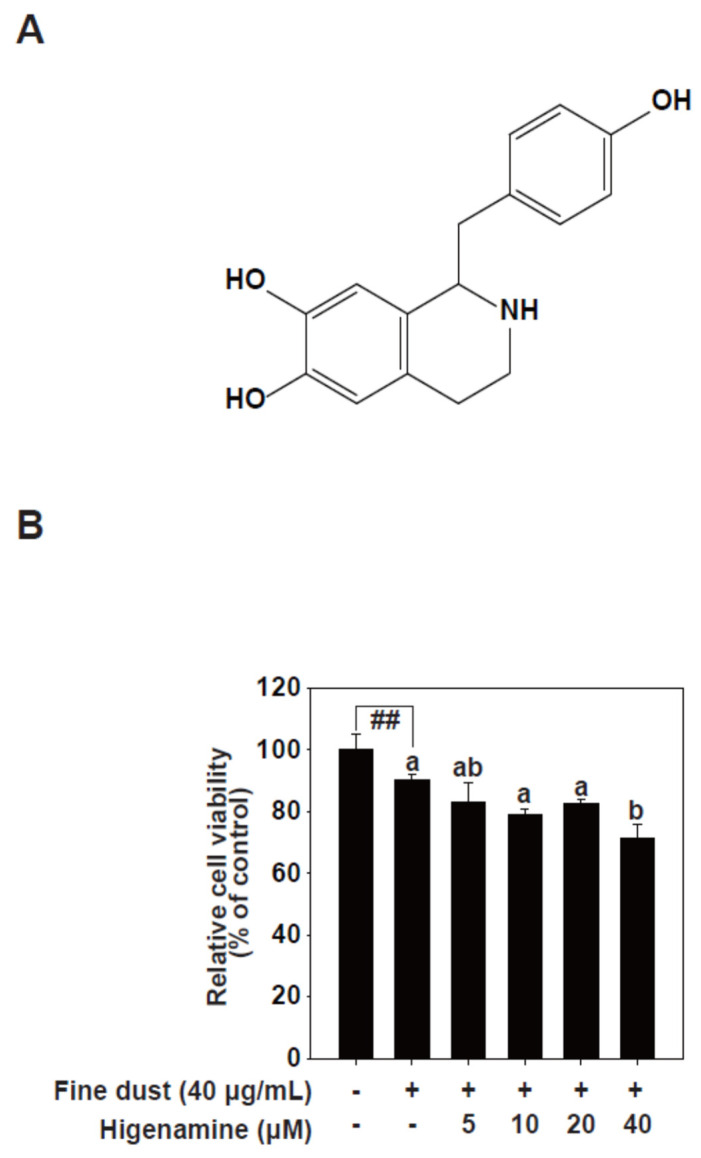
Effect of higenamine on HaCaT cell viability. (**A**), Chemical structure of higenamine. (**B**), Effect of higenamine on HaCaT cell viability. HaCaT cells were pretreated with higenamine at the indicated concentrations for 1 h, before exposure to fine dust (40 μg/mL). After 24 h, cell viability was measured using an MTT assay. Data (n = 5) are shown as the mean ± SD. Means with letters (a–b) in a graph indicate significant differences compared with control cells. The hashes (##) indicate a significant difference (*p* < 0.01) compared to untreated control.

Phytochemicals garnered scientific interest because of their historical use in traditional medicine [16]. *Aconitum carmichaelii*, native to East Asia and East Russia, comprises flowers, leaves, stems, roots, and seeds [17]. All parts of Aconitum were used in traditional medicine to reduce fever, alleviate asthma, and as sedatives [18,19]. Higenamine (Figure 1A), a plant alkaloid derived from the roots of Aconitum, was long used as a heart tonic. Higenamine increases the heart rate, promotes blood circulation, and induces vasodilation in muscles without raising blood pressure [20]. In animal studies, it was used as a β-2-adrenoreceptor agonist to treat asthma and alleviate respiratory distress [21,22,23]. Higenamine also demonstrated the potential to prevent and reduce blood clotting in animal models [24,25,26]. Some studies suggested that higenamine can reduce platelet aggregation and serve as a cardiotonic agent [27,28]. Although higenamine is sold as a dietary supplement for muscle building because of its vasodilatory effects, it is prohibited in professional sports due to its potential doping effects [29]. Despite the potential benefits of higenamine, its effect on fine-dust-induced skin aging is still unclear. In the current study, we explored the impact of higenamine on fine-dust-triggered MMP-1 production in human keratinocytes (HaCaT cells) in vitro, and clarified the molecular processes involved.

## 2. Results

### 2.1. Higenamine Attenuates Fine-Dust-Induced MMP-1 Protein Expression

**Figure 2:**
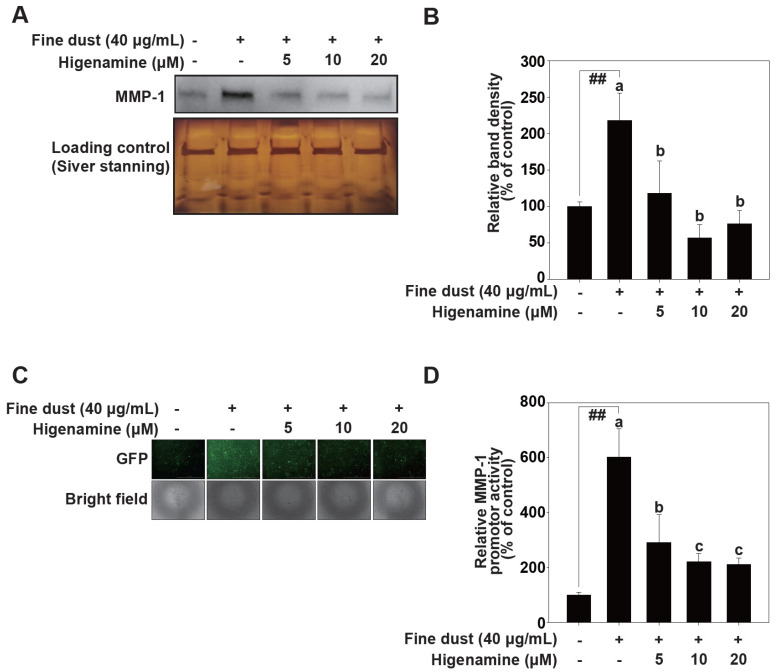
Higenamine reduces fine-dust-induced MMP-1 protein expression and MMP-1 promoter activity in HaCaT cells. (**A**,**B**), Effect of higenamine on MMP-1 expression on HaCaT cells. Higenamine was added to HaCaT cells for 1 h before being exposed fine dust (40 μg/mL) on HaCaT cells. After 24 h of fine dust treatment, protein expression was analyzed by Western blotting as described in the material and methods. Means with different letters (a–c) within a graph were significantly different from each other at *p* < 0.05. The hashes (##) indicate a significant difference (*p* < 0.01) compared to untreated control. Loading control was determined by silver gel staining. (**C**,**D**), MMP-1 promoter activity was measured using GFP gene reporter assay. HaCaT transduced with an MMP-1 reporter plasmid were pretreated with higenamine at the indicated concentrations for 1 h and were then further treated with fine dust. Cell extracts were collected after 24 h. MMP-1 promoter activity was measured using a the GFP gene reporter assay. Data (n = 3) represent the mean values ± SE. Means with letters (a–c) in a graph are significantly different from each other at *p* < 0.05 (## *p* < 0.01), relative to the control cells.

Collagen, a crucial structural protein in the ECM of connective tissue, is degraded by collagenase in aging skin [12]. Fine dust stimulates collagenase activity, leading to reduced collagen levels [30]. MMP-1 is an enzyme that holds significant importance for skin health [10]. Primarily involved in the degradation of collagen, which is the most abundant protein in the skin, MMP-1 aids in the natural process of skin regeneration and repair. However, it is crucial to maintain a balance, as excessive degradation of collagen due to increased activity of MMP-1 can lead to a loss of skin elasticity and contribute to the formation of wrinkles [11]. Therefore, MMP-1 is a major biomarker of skin aging. Our results showed that higenamine effectively decreased fine-dust-induced MMP-1 protein expression in a concentration-dependent manner in HaCaT cells (Figure 2A,B). We next investigated MMP-1 transcriptional activity. GFP reporter gene assays showed that fine dust increased MMP-1 promoter activity levels, and higenamine treatment lowered this transcriptional activity (Figure 2C,D).

### 2.2. Higenamine Modulates Fine-Dust-Induced AP-1 and NF-κB Transactivation

**Figure 3:**
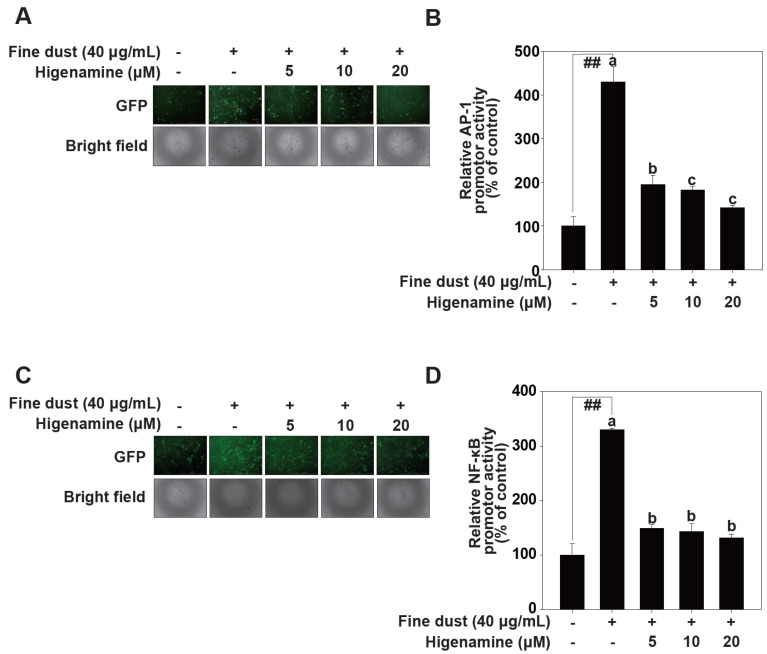
Higenamine reduces fine-dust-induced signaling by inhibiting AP-1 and NF-κB transactivation in HaCaT cells. Transactivation of AP-1 (**A**,**B**) and NF-κB (**C**,**D**) was measured using a GFP reporter gene assay. HaCaT cells transduced with an AP-1 or NF-κB reporter plasmid were pretreated with the indicated concentrations of higenamine for 1 h, and then further treated with fine dust. After 24 h and transactivation was measured as described in Materials and Methods. Data (n = 3) represent the mean values ± SE. Means with letters (a–c) in a graph indicate significant differences from each other at *p* < 0.05 (## *p* < 0.01), relative to the control cells.

Given that fine-dust-induced MMP-1 transcription depends on AP-1 and NF-κB transcription factors [30], we examined AP-1 and NF-κB transactivation levels in HaCaT cells treated with higenamine. Higenamine treatment also decreased fine-dust-induced AP-1.

(Figure 3A,B) and NF-κB transactivation (Figure 3C,D). Our results suggest that higenamine inhibits fine-dust-induced MMP-1 transcription by suppressing AP-1 and NF-κB transactivation.

### 2.3. Higenamine Impedes Fine-Dust-Induced MAPK Phosphorylation in HaCaT cells and HDFs

**Figure 4:**
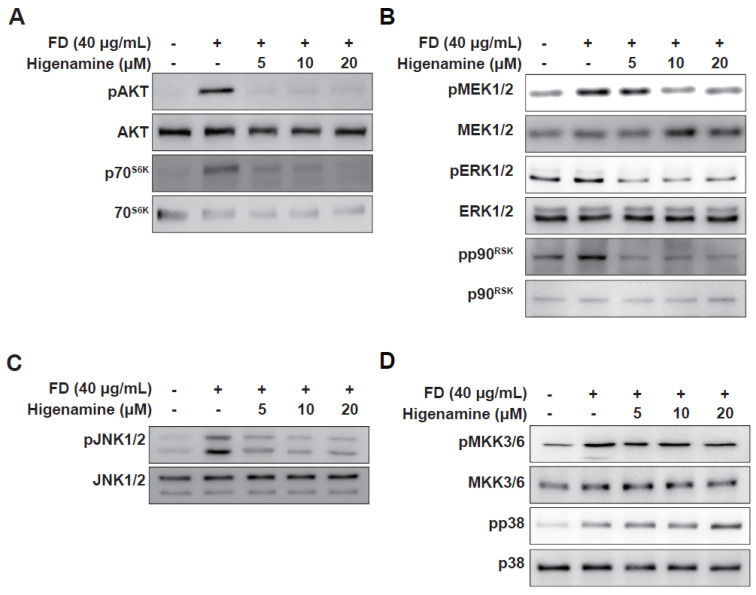
Inhibitory effect of higenamine on fine-dust-induced signaling pathways. Effect of higenamine on fine-dust-induced phosphorylation of (**A**) Akt-p70S6K, (**B**) MEK1/2-ERK1/2-p90RSK, (**C**) JNK1/2, and (**D**) MEK3/6-p38. Following higenamine pretreatment and fine dust exposure, the cells were lysed and phosphorylated, and total protein expression was determined by Western blotting with the indicated antibodies.

We investigated the effects of higenamine on the MAPK and Akt pathways, which, when activated, can lead to the activation of transcription factors AP-1 and NF-κB [10]. Fine dust exposure significantly increased the phosphorylation levels of several kinases (Figure 4). Higenamine treatment selectively hindered the phosphorylation of Akt-p70S6K, MEK1/2-ERK1/2-p90RSK, and JNK1/2 but not MKK3/6-p38 (Figure 4D). Our findings indicate that higenamine inhibits MMP-1 expression through the MEK1/2-ERK1/2-p90RSK, JNK1/2, and Akt-p70S6K pathways.

### 2.4. Higenamine Alleviates Fine-Dust-Induced Oxidative Damage in HaCaT Cells

**Figure 5:**
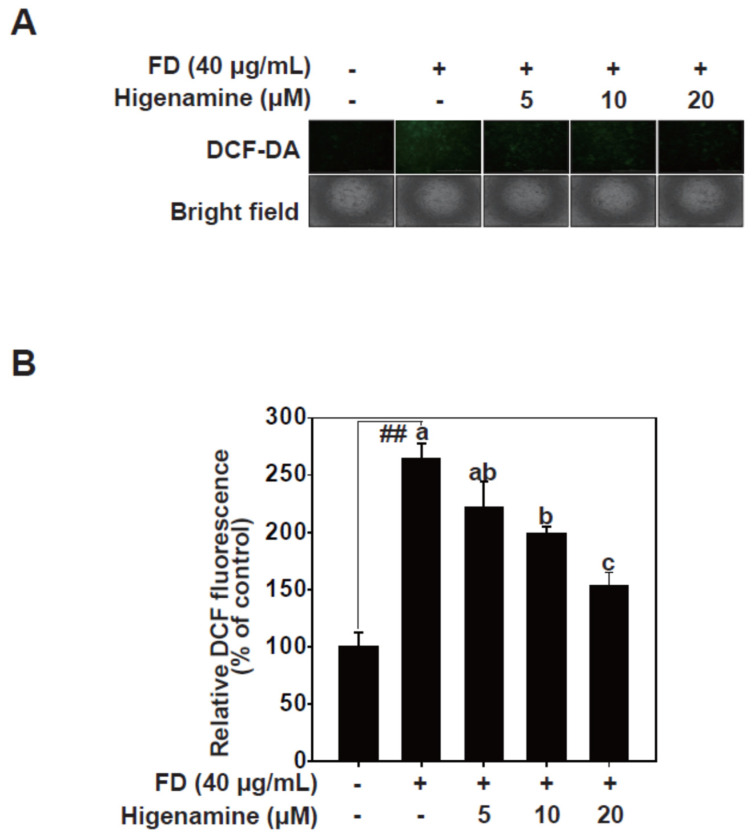
Higenamine reduces fine-dust-induced oxidative damage in HaCaT cells. (**A**,**B**), Images and quantification of the effects of fine-dust-induced ROS reduction by higenamine were measured using a DCF-DA fluorescence-based assay. Means with different letters (a–c) within a graph indicate significant differences from each other, with *p* < 0.05. The hashes (##) indicate a significant difference (*p* < 0.01) compared to the untreated control.

Exposure to fine dust can lead to an increase in oxidative stress, which is characterized by the generation of ROS [6]. ROS comprises unstable oxygen molecules that, when produced excessively, have the potential to inflict cellular damage. The generation of ROS plays a pivotal role in the activation of signaling pathways [30]. ROS interacts with signal transduction molecules, influencing a plethora of biological responses including inflammation and apoptosis, amongst various cellular functions.Oxidative stress, primarily associated with fine dust, impairs various skin functions due to ROS. In our study, we measured intracellular ROS levels and assessed cell damage caused by fine dust exposure [31]. Fine dust treatment led to increased intracellular ROS in HaCaT cells. Higenamine significantly reduced intracellular ROS levels in fine-dust-stimulated HaCaT cells in a dose-dependent manner (Figure 5). Our results suggest that higenamine effectively diminishes ROS and may provide relief for skin aging associated with fine dust exposure.

## 3. Discussion

Our study aimed to understand the protective properties of higenamine against skin aging, primarily induced by the exposure to fine dust. The results we obtained indicate that Higenamine significantly mitigates the production of MMP-1 triggered by fine dust. These proteins, MMP-1, contribute largely to the degradation of collagen and elastin, two significant components maintaining the structure and firmness of the skin [32]. Consequently, it is plausible to infer that the protective impact of higenamine may be attributed to its potential to curb the activity of these MMP, consequently preventing the breakdown of the extracellular matrix and preserving skin integrity [33].

Oxidative stress is a pivotal contributor to skin damage and premature aging, particularly when caused by exposure to fine dust. Fine dust, when it comes in contact with the skin, generates reactive oxygen species (ROS), leading to various skin ailments, including inflammation and expedited aging [30,34]. These minute particles of dust have the potential to penetrate deep layers of skin, causing direct damage as well as secondary damage, primarily through ROS generation [33]. The repercussions of this process are severe and encompass DNA damage, protein damage, and lipid peroxidation, all of which contribute to visible signs of skin aging and increased susceptibility to skin diseases [6].

Antioxidants have the inherent ability to neutralize ROS, thereby limiting their damaging effects. This led to the increased development of antioxidant-rich skincare products that aim to counter the impact of environmental pollutants like fine dust [3,33]. Our study demonstrated that higenamine, apart from its other beneficial impacts, can effectively act as an ROS scavenger, enhancing the antioxidant defense system, maintaining skin homeostasis, and forestalling premature skin aging caused by pollutants. Furthermore, our research revealed that Higenamine can significantly reduce fine-dust-induced MMP-1 protein expression and transactivation. It is noteworthy that higenamine also inhibited fine-dust-induced AP-1 and NF-κB transactivation [10,11]. These transcription factors play a central role in MMP-1 transcription and are known to be key mediators of inflammatory responses. Additionally, higenamine treatment noticeably curbed the generation of ROS, which are the primary contributors to oxidative stress and inflammation.

Higenamine effectively inhibited fine-dust-induced signaling in Akt, ERK1/2, and JNK1/2. However, it did not suppress the phosphorylation of p38. These results suggest that within the (MAPK) pathway, p38 protein kinase may be less responsive to ROS. ROS were generated by exposure to fine dust, and while substantial inhibition was observed in other MAPK components such as Akt, ERK1/2, and JNK1/2 with higenamine treatment, p38 protein kinase does not seem to exhibit the same level of influence. This pattern indicates that p38 protein kinase may not be significantly impacted by ROS in the signaling induced by fine dust exposure [35]. Such an observation highlights the complexity of the physiological responses to fine dust, where signaling is not purely linear but involves complex interactions between ROS and various molecular entities across different signaling pathways [11]. This complexity makes it challenging to predict how the activation of one pathway may influence the activation of others. Therefore, to fully comprehend the effects and mechanism of higenamine, further research encompassing various signaling pathways, including p38 protein kinase, is warranted [10]. Such investigations could enhance our understanding and potentially improve the preventive capacity of higenamine against skin damage induced by fine dust.

Historically, higenamine, also known as norcoclaurine, is a naturally occurring compound derived from various plant sources such as Nandina domestica (sacred bamboo), Annona squamosa, and Nelumbo nucifera (lotus seeds). These plants were part of traditional medicine, particularly in East Asia, suggesting the potential therapeutic properties of higenamine [36]. Some studies proposed potential positive influences of higenamine on cardiovascular health, weight management, and respiratory health [21,22,23]. This is why the substance is sometimes abused by athletes. The World Anti-Doping Agency (WADA) bans any substances that could provide an unfair competitive advantage in any sport. Therefore, higenamine was included in WADA’s prohibited substance list since 2017. However, the scientific evidence for higenamine’s performance-enhancing effects is insufficient and there do not appear to be significant safety issues for general consumption. Thus, unless you are an athlete, there should be no problem using it [22].

In conclusion, our study provided substantial evidence of the protective effects of higenamine against fine-dust-induced skin aging. This can be mainly attributed to its MMP-1 inhibitory properties and its potential to mitigate ROS generation caused by fine dust exposure. These findings suggest that higenamine could serve as a promising candidate for the development of skincare products aimed to combat the adverse effects of environmental pollutants on skin health.

## 4. Materials and Methods

### 4.1. Chemicals and Materials

High Dulbecco’s modified Eagle medium (DMEM), Penicillin–streptomycin solution and Trypsin-EDTA Solution were purchased from Welgene (Gyeongsan, Republic of Korea). Fetal bovine serum (FBS) was purchased from Atlas Biologicals (Fort Collins, CO, USA). Standardized fine dust (PM10-like) (European Reference Material ERM-CZ100) was purchased from European Commission’s Joint Research Centre (Brussels, Belgium). The MMP-1 antibody was purchased from R&D Systems Inc. (Minneapolis, MN, USA). Antibodies against the phosphorylated extracellular-signal regulated kinase (ERK) 1/2 at the Thr202/Tyr204 residues, total ERK1/2, total Akt, and total c-Jun N-terminal kinase 1 (JNK1) were obtained from Santa Cruz Biotechnology (Santa Cruz, CA, USA). Other antibodies were purchased from Cell Signaling Biotechnology (Beverly, MA, USA). The lentiviral expression vectors including pGF-AP1-mCMV-EF1-Puro, pGF-NF-κBmCMV-EF1-Puro, pGF-MMP-1-mCMV-EF1-puro (System Biosciences, CA, USA), packaging vectors including pMD2.0G and psPAX were purchased from Addgene Inc. (Cambridge, MA, USA). ECL prime Western blotting detection reagent was purchased from Amersham (Little Chalfont, UK).

### 4.2. Cell Culture

HaCaT Keratinocytes were included from CLS Cell Lines Services GmbH (Heidelberg, Germany) and cultured in DMEM with 10% FBS and 1% antibiotics at 37 °C, 5% CO2. Cells were periodically subcultured, and the cells in log phase were used for the experiments.

### 4.3. Preparation of Fine Dust

Fine dust was prepared at a concentration of 25 mg/mL mixed with dimethyl sulfoxide (DMSO). It was sonicated for 40 min to avoid agglomeration of fine dust. In this study, the stock of fine dust was diluted with serum-free DMEM and used in experiments.

### 4.4. Cell Viability

3-[4,5-dimethylthiazol-2-yl]-2,5-diphenyl tetrazolium bromide (MTT) assay was performed to evaluate the cytotoxicity of fine dust and/or higenamine. HaCaT cells were cultured on a 96-well plate and treated fine dust and/or higenamine for 24 h. After time, the cells were treated with MTT solution (0.45 mg/mL) and incubated at 37 °C for 2 h. After the medium was removed, the formazan dye in the cells was solubilized in DMSO (200 μL). The dye solution was transferred to the new 96-well plate for 100 μL and absorbance was measured at 570 nm using microplate reader (Biotek, VT, USA).

### 4.5. ROS Measurement

2’,7’-dichlorofluorescin-diacetate (DCF-DA) assay was performed to measure the reactive oxygen species (ROS) of fine dust and/or higenamine. HaCaT cells were cultured black 96-well plate and treated 100 μM DCF-DA solution for 30 min. Remove DCF-DA solution and wash cells with Hank’s Balanced Salt Solution (HBSS). Incubate plate for 30 min, remove HBSS and add fine dust and/or higenamine diluted in HBSS for 2 h. Conversion of DCF-DA into DCF took pictures and measured at an extinction of 485 nm and an emission of 530 nm using Cell Imaging Multi-Mode Reader (Biotek, VT, USA).

### 4.6. Western Blot

HaCaT cells were incubated in serum-free DMEM for 24 h and treated with higenamine (5, 10, 20 μM) for 1 h and treated fine dust. For extracellular matrix proteins, the cultured medium was collected. For intracellular proteins, cell lysates were prepared using cell lysis buffer (50 mM tris-HCl (pH 8.0), 0.15 M NaCl, 1% NP-40, 0.1% SDS, 0.5% deoxycholate, 1 mM dithiothreitol, 1 mM phenylmethylsulfonyl fluoride 1 mM Na_3_VO_4_). Both the cultured medium and cell lysates were harvested on ice and centrifuged at 13,000 rpm for 10 min, and supernatant was collected. Protein concentrations were measured using a protein assay kit (Bio-Rad, Hercules, CA, USA). The proteins were electrophoretically separated using an 8%, 10% SDS-polyacrylamide gel and transferred onto 0.2 μm PVDF membranes (Little Chalfont, UK). The membrane was blocked in 5 % non-fat milk or 5% bovine serum albumin (BSA) for 1 h. The membrane was incubated with specific primary antibody for 4 °C overnight. After washing the membrane, the HRP-conjugated secondary antibody (Santa Cruz) was combined on membrane for room temperature 1 h. Protein bands were detected using an ECL prime Western blotting detection kit (GE Healthcare, Chicago, IL, USA). The Western blot data were analyzed using quantitative analysis in Image Studio software 5.5.4 (LI-COR, Lincoln, NE, USA).

### 4.7. GFP Reporter Gene Assay

pGF-AP-1-mCMV-EF1-Puro vector, pGF-NF-κB-mCMV-EF1-Puro, and pGFMMP-1-mCMV-EF1-puro vector with the packaging vectors (psPAX and pMD2.0G) were, respectively, transfected into HEK293T cells using jetPEI, following the manufacturer’s instructions. The transfection medium was changed 24 h after transfection. The cells were cultured for another 36 h and the viral particles were prepared using a syringe filter (0.45 μm). HaCaT cells were infected with 8 μg/mL of polybrene (EMD Millipore, MA, USA) overnight. The cell culture medium was replaced with fresh growth medium, and the cells could recover for 24 h, before they were subjected to 2 mg/mL puromycin (Sigma, MO, USA) selection for 36 h. The HaCaT cells starved in serum-free media for 24 h. Then, the cells were treated with fine dust and/or higenamine. Fluorescence generated in the cells was measured at 469–525 nm using a fluorescence meter.

### 4.8. ROS Measurement

2’,7’-dichlorofluorescin-diacetate (DCF-DA) assay was performed to measure the reactive oxygen species (ROS) of fine dust and/or higenamine. HaCaT cells were cultured black 96-well plate and treated 100 μM DCF-DA solution for 30 min. Remove DCF-DA solution and wash cells with Hank’s Balanced Salt Solution (HBSS). Incubate plate for 30 min, remove HBSS, and add fine dust and/or higenamine diluted in HBSS for 2 h. Conversion of DCF-DA into DCF was measured at an extinction of 485 nm and an emission of 530 nm and obtained images using Citation 5 (Biotek, VT, USA).

### 4.9. Statistical Analysis

All experiments were repeated three times, and all data were analyzed using SPSS Statistics software v.18.0 (IBM, New York, NY, USA). The difference between the control group and the fine-dust-treated control group was evaluated by Student’s *t*-test. The differences between the fine-dust-treated groups were compared using Duncan’s multi-range test and one way ANOVA. A probability value of less than 0.05 was regarded as the criterion for statistical significance.

## Data Availability

There is no available data.
